# Volcano‐Inspired Dual‐Carbon Network Aerogel for High‐Performance Solar Evaporation With Edge‐Directed Salt Crystallization and Recovery

**DOI:** 10.1002/advs.74823

**Published:** 2026-03-12

**Authors:** Shuyue Feng, Yongpeng Wang, Mengzhu Liu, Xin Wang, Tao Jia, Haibo Zhang, Haoyue Wu, Yuan Xu, Linghui Kong

**Affiliations:** ^1^ College of Materials Science and Engineering Jilin University of Chemical Technology Jilin China; ^2^ College of Chemistry and Chemical Engineering China University of Petroleum (East China) Qingdao China; ^3^ Key Laboratory of Forest Plant Ecology, Ministry of Education, Engineering Research Center of Forest Bio‐Preparation, College of Chemistry, Chemical Engineering and Resource Utilization Northeast Forestry University Harbin China; ^4^ College of Chemistry Jilin University Changchun China

**Keywords:** aerogels, desalination, dual‐carbon networks, marangoni effect, salt crystallization, solar‐driven

## Abstract

The escalating global freshwater shortage demands sustainable and energy‐efficient desalination solutions. Here, a volcano‐inspired dual‐carbon‐network aerogel (CFCA) that couples hierarchical fluidic channels with multiscale thermal management for high‐performance solar desalination is developed. The CFCA features interconnected primary, branching, and microporous conduits within a carbon‐black aerogel, reinforced by dispersed short carbon fibers to form continuous capillary pathways for rapid water transport and vapor release. A continuous outer carbon‐fiber shell acts as a heat‐confining layer and thermal reservoir while directing salt migration toward the edges. The conical geometry generates radial temperature gradients that induce Marangoni convection, enabling spontaneous salt expulsion and edge‐localized crystallization without blocking the photothermal surface. Benefiting from these synergistic effects, the CFCA delivers an ultrahigh evaporation rate of 4.08 kg m^−2^ h^−1^ and a solar‐to‐vapor efficiency of 95.8% under 1 sun. It also maintains an evaporation rate of 2.63 kg m^−2^ h^−1^ in 20 wt.% brine with a salt recovery efficiency of 44.2%, and achieves an average rate of 3.00 kg m^−2^ h^−1^ during seven‐day operation in 3.5 wt.% saline water. This work establishes a comprehensive design strategy integrating efficient photothermal conversion, guided mass transport, and recoverable salt crystallization for scalable solar‐driven water purification and salt resource recycling.

## Graphical abstract

A volcano‐inspired dual‐carbon aerogel featuring hierarchical conduits and an outer carbon‐fiber shell directs rapid water transport, confines heat, and enables directional salt migration. Radial temperature gradients in the conical structure trigger Marangoni‐driven edge‐localized salt crystallization while maintaining an unobstructed photothermal interface, achieving ultrahigh evaporation rates of 4.08 kg m^−^^2^ h^−^^1^ under 1 sun and 2.63 kg m^−^^2^ h^−^^1^ in 20 wt.% NaCl with 44.2% salt recovery.

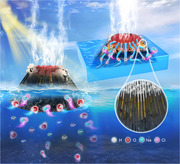

## 1 Introduction

The escalating global freshwater crisis, driven by population expansion, industrialization, and climate anomalies, has underscored the urgent need for sustainable water purification technologies [1, 2, 3]. Among various approaches, seawater desalination stands out as a viable solution; yet conventional methods—such as multi‐effect distillation (MED), multi‐stage flash (MSF), and reverse osmosis (RO)—remain hindered by excessive energy demand, high capital investment, and environmental burdens arising from brine discharge [4, 5, 6]. In this context, solar‐driven interfacial evaporation (SIE) has emerged as a disruptive paradigm, enabling low‐cost and carbon‐neutral desalination through localized solar‐thermal conversion at the air–water interface [7, 8, 9, 10].

The performance of SIE systems depends critically on the interplay between photothermal materials and architectural design [11, 12, 13, 14]. Ideal photothermal absorbers should exhibit broadband light absorption, efficient photothermal conversion, robust water transport, and long‐term salt tolerance [15, 16, 17, 18, 19, 20]. The photothermal materials for solar steam generators mainly include carbon‐based materials [12, 21, 22, 23, 24, 25, 26], metal semiconductor materials [27, 28, 29, 30, 31, 32], metal nanoparticle materials [33, 34], and conjugated polymer materials [35, 36]. Carbon‐based materials such as carbon black [37, 38], carbon nanotubes [39], and carbon fibers [23, 24] have been extensively investigated owing to their intrinsic optical absorptivity, chemical stability, and cost‐effectiveness [40]. Among the broad family of carbon‐based photothermal materials, fiber‐reinforced carbon aerogels (CAs) stand out as a compelling class of multifunctional scaffolds. These materials combine ultralight density, hierarchical porosity and tunable thermophysical behavior with robust mechanical integrity. By integrating one‐dimensional carbon fibers into the three‐dimensional aerogel matrix, one can establish continuous conductive pathways and interconnected capillary networks, thereby simultaneously promoting broadband solar absorption, rapid capillary water transport and thermal confinement [41, 42]. For example, Yan J. et al. reported a carbon nanofiber‐reinforced carbon aerogel achieving an evaporation rate of 3.82 kg m^−2^ h^−1^ with 95.5% efficiency by enhancing water transport and thermal management [43]. Recent studies of carbon fiber‐reinforced carbon aerogels demonstrate how such architectures yield improved solar‐driven interfacial evaporation performance via enhanced heat localization and mass transport regulation [44].

Beyond material composition, structural engineering plays a pivotal role in enhancing light capture, vapor release and salt‐handling. However, conventional aerogel evaporators often experience uncontrolled heat dissipation and salt accumulation, which compromise both evaporation rate and durability. To overcome these constraints, biomimetic and three‐dimensional (3D) architectures have recently drawn intensive attention for improving photon trapping, vapor diffusion, and salt management [36, 45, 46, 47, 48, 49]. Zhao. et al. developed a tree‐inspired structurally graded aerogel featuring root‐like microchannels and surface‐aligned pores, which collectively govern water uptake, downward salt transport, and heat localization, achieving stable solar‐driven evaporation rates of 2.09 kg m^−2^ h^−1^ in 3.5 wt.% NaCl and 1.94 kg m^−2^ h^−1^ in 20 wt.% NaCl without salt crystallization [50]. Ma et al. developed a biomimetic 3D aerogel (SrGAE) with vertically arranged channels inspired by seagrass vascular bundles, which govern water transport, salt discharge, and heat management, achieving a stable solar‐driven evaporation rate of 3.7 kg m^−2^ h^−1^ and a solar‐vapor conversion efficiency of 106 % in 20 wt.% brine under one‐sun illumination [51]. Hu et al. developed a lotus‐leaf inspired Janus‐structured biomimetic aerogel evaporator with vertically aligned nanofiber networks and asymmetric wettable surfaces, which govern water transport, salt rejection, and light absorption, achieving a solar‐driven evaporation rate of 2.78 kg m^−2^ h^−1^ and stable performance in 20 wt.% brine under one‐sun illumination [52]. Jin et al. developed an iceberg‐inspired inverted solar water generator (ISWG) comprising a photothermal‐coated thermoelectric layer, bilayer all‐fiber nonwoven fabric, and passive cooling vapor condenser, which governs heat management, vapor collection, and electricity generation, achieving a vapor collection rate of 1.02 kg m^−2^ h^−1^ and a power output of 0.47 W m^−2^ under one‐sun illumination [53]. Despite these advancements, achieving simultaneous heat confinement, efficient water evaporation, and controllable salt expulsion remains a formidable challenge, mainly due to the intrinsic coupling and trade‐offs among heat localization, water transport, and salt diffusion in porous evaporators [54, 55, 56].

Inspired by the natural architecture of volcanoes, we developed a dual‐carbon‐network aerogel (CFCA) that mimics both the hierarchical morphology and the functional dynamics of volcanic systems. The aerogel features a multi‐level channel network, including primary conduits, branching channels, and microporous sub‐passages, analogous to the magma transport system of a volcano. Within this framework, dispersed short carbon fibers are uniformly embedded throughout the carbon‐black aerogel matrix, forming a highly interconnected capillary network. This design enables efficient water replenishment through capillary pumping and rapid vapor escape via interconnected pores, thereby maintaining continuous mass transport during solar irradiation [57]. The combination of the internal porous conduits and short carbon fibers effectively minimizes vapor blockage and ensures a steady water supply toward the evaporation interface. Additionally, a bowl‐shaped upper surface mimicking the volcanic crater assists in vapor collection and light trapping, amplifying solar absorption and local vapor pressure.

Surrounding this inner architecture, a continuous carbon‐fiber outer shell is constructed to emulate the volcanic crust. This layer serves multiple synergistic roles that it acts as a thermal barrier that confines heat within the evaporation zone and a heat‐storage medium that buffers temperature fluctuations [58, 59]. Moreover, this peripheral carbon‐fiber network provides directional pathways for salt and water migration, facilitating the guided outward transport of saline solution toward the rim. Notably, the volcano‐like conical morphology introduces establishes a temperature gradient, which triggers a pronounced Marangoni convection field, driving water and solute transport from the central hot zone toward the peripheral region. This flow behavior enables spontaneous salt expulsion, whereby salt ions and crystals migrate and crystallize preferentially along the upper rim of the aerogel cone. Such edge‐localized crystallization not only allows for in‐situ salt harvesting but also maintains an unobstructed photothermal surface, preserving the effective light‐absorbing and evaporative area [60, 61]. Therefore, the CFCA system accomplishes the dual objectives of continuous desalination and salt recovery without compromising solar energy conversion efficiency.

Benefiting from the synergistic coupling of the dual‐carbon networks, multi‐channel volcanic conduits, and directional Marangoni‐driven salt migration, the CFCA evaporator achieves an ultrahigh evaporation rate of 4.08 kg m^−2^ h^−1^ under 1 sun illumination, corresponding to a remarkable solar‐to‐vapor conversion efficiency of 95.8%. Even when challenged with 20 wt.% saline solution, the system still maintains a high evaporation rate of 2.63 kg m^−2^ h^−1^ under 1 sun illumination, while enabling directional salt crystallization exclusively at the rim with a salt recovery efficiency of 44.2%. Furthermore, during continuous operation in 3.5 wt.% saline water for seven days, the device preserves an average evaporation rate of 3.00 kg m^−2^ h^−1^, demonstrating outstanding long‐term desalination stability. These synergistic effects collectively establish a comprehensive paradigm for sustainable, high‐efficiency solar‐driven water purification and salt resource recycling, highlighting the practical potential of the volcano‐inspired dual‐carbon aerogel architecture in scalable solar desalination technologies.

## 2 Experimental Section

### 2.1 Preparation of Volcanic Peripheral Frame

First, 7 g PVA powders were dissolved in 100 g deionized (DI) water at 120°C for 2 h. Then, arrange the carbon fibers in an orderly manner and uniformly coat the surface of the carbon fibers with the above PVA solution. Place the sample in an oven at 80°C for 24 h to obtain a carbon fiber membrane. Next, curl and shape the carbon fiber membrane to construct the outer framework of a volcanic structure, thereby achieving a volcanic‐shaped carbon fiber shell structure.

### 2.2 Fabrication of CFCA

**FIGURE 1:**
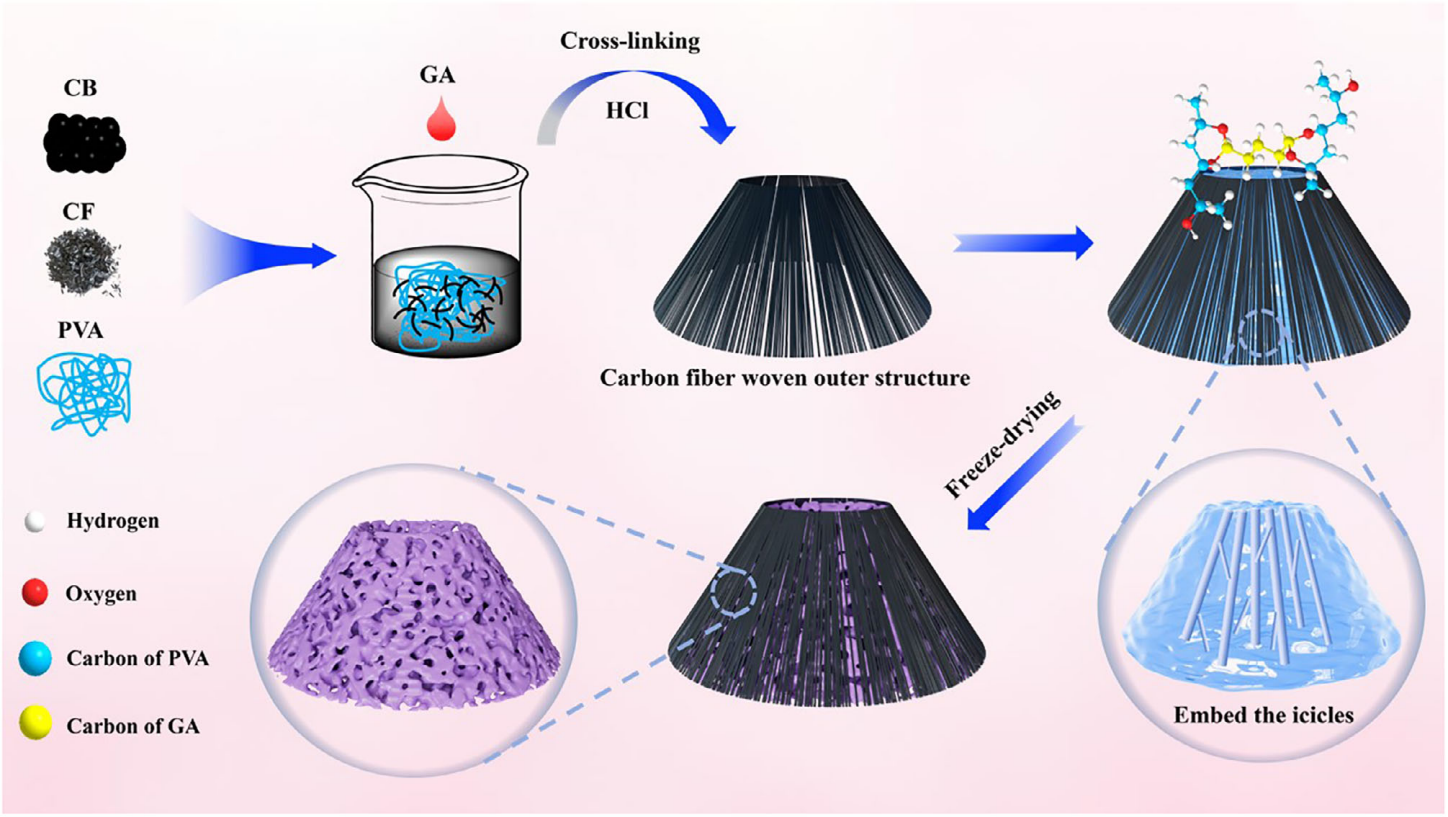
Schematic illustration of the fabrication of series of CFCA.

1.5 g of polyvinyl alcohol (PVA) was added to 48.5 mL of deionized water and stirred for 2 h on a magnetic stirrer at 100°C, resulting in a 3 wt.% polyvinyl alcohol solution. To this polyvinyl alcohol solution, 1.5 g of carbon black and 0.15 g of short‐cut carbon fibers were added, and the mixture was subjected to mechanical stirring and ultrasonic dispersion treatment to obtain a uniformly dispersed carbon fiber/carbon black/polyvinyl alcohol solution. Glutaraldehyde (GA) was used as a chemical crosslinking agent for the carbon fiber/carbon black/polyvinyl alcohol solution, and 50 mL of the solution was poured into a volcanic‐shaped carbon fiber shell, followed by the addition of 0.6 mL of glutaraldehyde. Mechanical stirring and ultrasonic treatment were further applied to promote the acetal reaction between glutaraldehyde and polyvinyl alcohol molecules, resulting in a carbon fiber/carbon black/polyvinyl alcohol acetal solution, to which a 1 mol L^−1^ hydrochloric acid solution was added to facilitate crosslinking. Subsequently, a volcanic‐shaped carbon fiber/carbon black/polyvinyl alcohol acetal hydrogel was obtained. To guide the formation of an internal channel structure, small ice pillars were embedded within the hydrogel to construct volcanic channels. The system was placed in a freeze‐drying apparatus, and after vacuum freeze‐drying for 36 h, a volcanic‐shaped carbon fiber/carbon black/polyvinyl alcohol acetal aerogel (CFCA) with a volcanic channel structure was obtained. The preparation process and mechanism are shown in Figure 1. The real optical photographs of the as‐fabricated CFCA evaporator are provided in Figure S1, which clearly demonstrate the volcano‐like macroscopic morphology.

For aerogels without a carbon fiber shell, they are designated as CFA. For aerogels that neither possess a carbon fiber shell nor disperse short‐cut carbon fibers internally, they are designated as CPA.

## 3 Results

### 3.1 Biomimetic Design of CFCA

**FIGURE 2:**
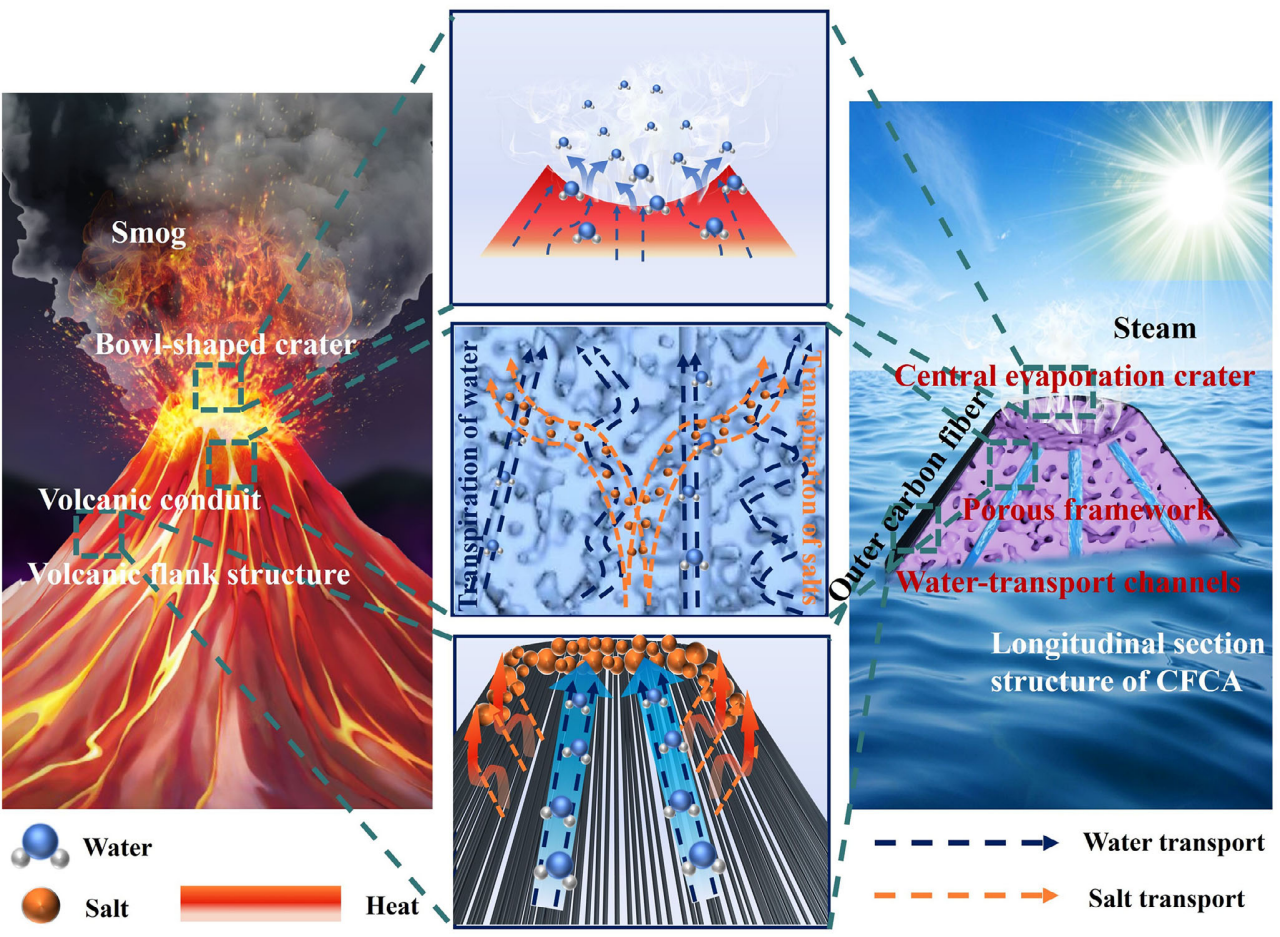
Schematic illustration of the volcano‐inspired CFCA evaporator. Schematics showing the water and mass transport mechanisms in volcano and CFCA.

Volcanic eruptions are caused by the increase in temperature of underground magma, which leads to an increase in pressure within the magma chamber. This pressure causes the rock layers to fracture, and fissures begin to extend along the strata. Magma and gases rise through these fissures, forming a volcanic conduit. During this ascent, the magma may encounter geological structures such as faults and fractures as it moves through various internal pathways of the volcano (central conduit, vent conduit, lateral conduit) and different geological layers. Throughout this process, the gases within the magma (particularly water vapor) are compressed and gradually released during the ascent, which enhances the gas emissions around the volcanic crater, resulting in significant gas discharge [62]. Here, we designed and manufactured a CFCA to simulate the transport system analogous to that in a volcano (Figure 2). CFCA is made from a mixture of external carbon fibers, internal short‐cut carbon fibers, carbon black, and polyvinyl alcohol, exhibiting excellent hydrophilicity and light absorption properties. CFCA has a truncated cone shape that is narrower at the top and wider at the bottom, with external walls radiating from top to bottom, resembling a volcanic structure. This design provides better stability and a larger water absorption contact area compared to flat surfaces. At the top of the CFCA, the external carbon fibers provide support, and during the freeze‐drying of the internal cross‐linked products, a shrinkage reaction occurs. Based on this, we constructed a bowl‐shaped structure resembling a volcanic crater, which offers a large surface area for solar energy absorption, thereby capturing solar energy more effectively and enhancing light absorption, significantly improving photothermal conversion performance. Consequently, moisture can be released from the top as water vapor through capillary action or evaporation, akin to the smoke emitted during a volcanic eruption. The internal porous structure of CFCA and the constructed channels are analogous to various types of channels within a volcano, combined with a highly interconnected capillary network formed by dispersed short carbon fibers. This design achieves efficient water replenishment through capillary pumping, allowing moisture to be transported upward from these channels via capillary forces. Due to the high thermal conductivity of the outer carbon fiber in the longitudinal direction, its external temperature is lower than that of the interior, which induces the Marangoni effect. This causes the liquid to flow from the high‐temperature region with low surface tension (interior) to the low‐temperature region with high surface tension (exterior), thereby reducing the salt concentration in the high‐temperature interior area and helping to prevent salt deposition in the evaporation zone. Similar to the channels within a volcano, the hydrophilic porous structure inside the CFCA and the constructed channels can efficiently transport water, continuously delivering it to the top. Finally, we utilized carbon fiber to simulate the shell of the volcano. The carbon fiber, with its high specific surface area, not only facilitates effective water transport but also possesses good thermal conductivity, allowing for rapid heat transfer to the exterior. This creates a temperature gradient with the internal structure, inducing the Marangoni effect, which effectively desalinate in high‐concentration saline water.

### 3.2 Structural Characteristics of CFCA

**FIGURE 3:**
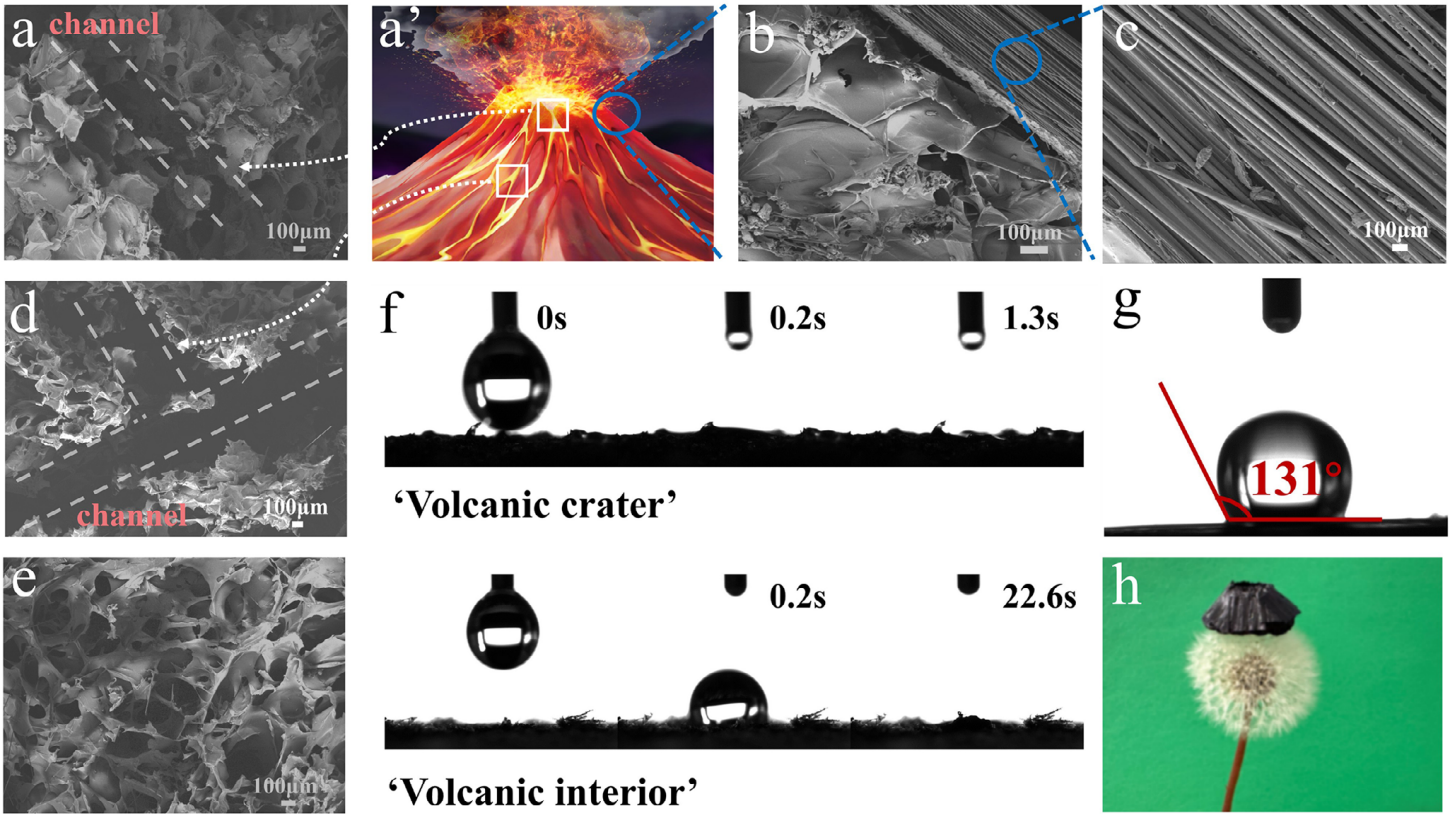
(a) Main channel SEM image inside CFCA. a' Volcanic profile structure diagram. (b) Peripheral SEM image of CFCA. (c) Enlarged SEM image of carbon fibers in the CFCA periphery. (d) SEM images of internal branch channels in CFCA. (e) SEM images of internal pores in CFCA. (f) Time sequence optical images of water droplet infiltration across different CFCA components. (g) Water contact angle on the surface of CFCA carbon fibers. (h) Optical photograph of CFCA placed on dandelions.

The SEM images of CFCA are shown in Figure 3a,d. By inserting ice pillars during the preparation, the internal structure of the CFCA successfully formed a main channel and branch channels resembling the interior of a volcano (Figure 3a'). As illustrated in Figure 3b,c, the outer carbon‐fiber shell mimics the volcanic outer crust and exhibits a compact and aligned fibrous structure, which enhanced mechanical stability and promoted directional water transport along the surface. The CFCA also constructed a three‐ dimensional pore structure through freeze‐drying, as shown in Figure 3e. It can be observed that short‐cut carbon fibers are also interspersed within, forming a highly interconnected capillary network that constructs water‐conducting bridges between pores, thereby providing an efficient transport pathway for the upward movement of moisture. To further quantitatively characterize the nanoscale pore features within the porous framework observed in the SEM images (Figure 3), nitrogen adsorption–desorption measurements were performed (Figure S2). As shown in Figure S2a, the adsorption–desorption isotherm of CFCA exhibits a typical type‐IV profile accompanied by a pronounced hysteresis loop, suggesting the existence of interconnected mesoporous domains. The gradual adsorption uptake at low‐to‐intermediate relative pressures indicates accessible internal pore surfaces, while the sharp increase near high relative pressure reflects the contribution of larger open voids, consistent with the macroscopic porous channels observed by SEM. The BET specific surface area of CFCA is determined to be 29.65 m^2^ g^−^^1^, confirming that the framework provides measurable internal interfacial area for capillary‐driven liquid transport. Furthermore, BJH pore size distribution analysis (Figure S2b) reveals that the pores are mainly concentrated in the 2–6 nm range, with a dominant peak located around 3–4 nm, demonstrating that mesopores constitute the primary nanoscale pore population. These mesoporous domains can generate strong capillary forces and enlarge the solid–liquid contact area, while the micrometer‐scale macroporous channels visualized in Figure 3 provide rapid pathways for water supply and vapor diffusion. Therefore, the combination of SEM observations (macroporous channels) and BET/BJH analyses (quantitative mesoporous characteristics) confirms that CFCA possesses a multiscale hierarchical porous architecture beneficial for efficient interfacial evaporation.

To evaluate the water transmission capacity, we first measured the contact angles of water on the top of the CFCA, the internal surface of the CFCA, and the outer carbon fiber surface. As illustrated in Figure 3g, 5 µL droplet of water was placed on both the top and the interior of the CFCA. The droplet on the top of the CFCA was completely absorbed within 1.3 s, while the droplet on the interior was fully absorbed in 22.6 s, which is slightly slower than the absorption rate on the top structure. This is due to the dispersion of short‐cut carbon fibers within the CFCA, which are hydrophobic in nature; thus, the water absorption rate in the interior of the CFCA is slightly slower than that on the top, providing conditions for subsequent salt exclusion. With the further incorporation of carbon fiber as the shell of the CFCA, as shown in Figure 3g, the water contact angle on the surface of the outer carbon fiber is measured at 131°, demonstrating its significant hydrophobicity. This makes its surface less susceptible to wetting by water. During the operation of the evaporator, moisture carries salt toward the edges during the evaporation process. Since carbon fiber does not adsorb moisture, brine is difficult to adhere to its surface, thereby achieving efficient salt rejection. As illustrated in Figure 3h, the CFCA can stably support itself on a dandelion, confirming that the aerogel we have prepared possesses extremely lightweight characteristics.

### 3.3 Photothermal, Water Management, and Mechanical Properties of CFCA

**FIGURE 4:**
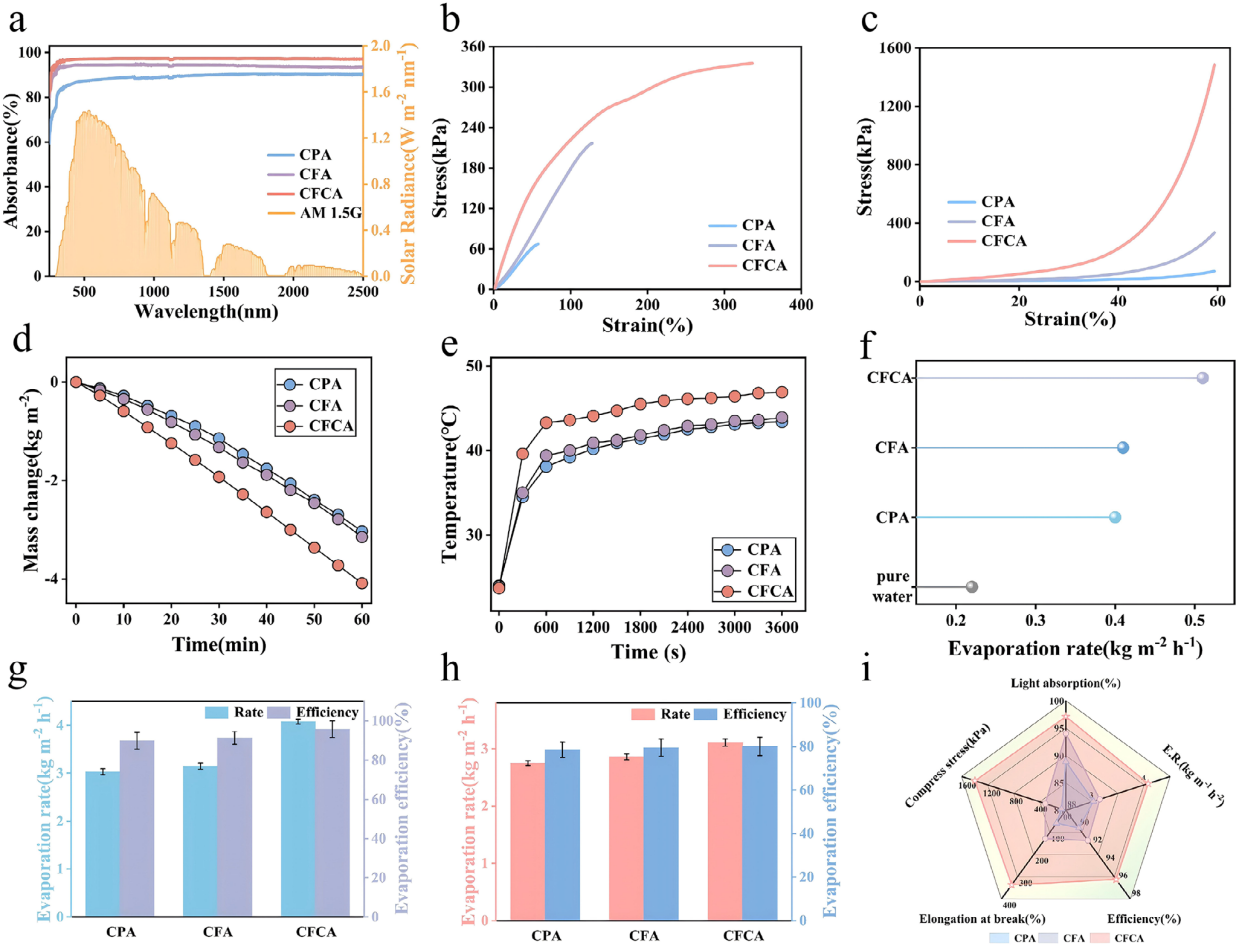
(a) The UV spectra of different samples, with the background showing the AM 1.5 solar spectrum. (b) Tensile properties of different samples. (c) Compression performance of different samples. (d) The water mass variation over time for different samples under 1 sun irradiation. (e) Surface temperature change for different samples under 1 sun irradiation. (f) The evaporation rates of different samples under dark conditions. (g) Comparison of the evaporation performance of different samples in pure water. (h) Comparison of the evaporation performance of different samples in 3.5 wt.% NaCl solution. (i) Comparison of the light absorption performance, compression performance, tensile property, evaporation rate, and evaporation efficiency of different samples.

To evaluate the solar capture capabilities of different samples, a UV‐Vis‐NIR spectrophotometer was employed. As shown in Figure 4a, the CFCA exhibits a broader solar light absorption capacity in the range of 200 to 2500 nm compared to CPA and CFA. This enhancement can be attributed to the inherent absorption characteristics of the peripheral carbon fiber's gray‐black surface, which improves the light absorption ability of CFCA. While the light absorption effects of CPA and CFA are similar, the presence of dispersed short‐cut fibers within CFA enhances its light absorption. The light absorption of the CFCA evaporator can reach up to 98% across the entire spectral range, demonstrating its excellent light capture capability. The internal porous network structure of CFCA allows sunlight to undergo multiple reflections within the material, thereby extending the light path and minimizing light scattering. To investigate the impact of the introduction of carbon fibers on the mechanical properties of the evaporator, a universal testing machine was employed to conduct tensile and compressive tests on CFCA, CFA, and CPA. As shown in Figure 4b, with the addition of short‐cut carbon fibers in the evaporator, the tensile strain increased from 57.84% to 128.01%, while the corresponding stress rose from 67.23 to 216.72 KPa. The tensile strength of CFA along the vertical axis is three times that of CPA, which is attributed to the fact that the short‐cut carbon fibers still possess good mechanical properties, providing support within the channels. Furthermore, with the incorporation of peripheral carbon fibers, the tensile strain of the evaporator significantly increased from 128.01% to 336.25%, and the corresponding stress escalated from 216.72 to 335.29 KPa. The tensile strength of CFCA along the vertical axis is 1.5 times that of CFA, a result of the high strength and stability of the carbon fibers. The compression curve shown in Figure 4c indicates that at a compressive strain of 60%, the stress values for CPA, CFA, and CFCA are 71.09, 333.52, and 1483.43 KPa, respectively. Compared to CPA and CFA, CFCA exhibits a significant improvement in compressive performance in the longitudinal direction, suggesting that the introduction of high‐strength and ductile carbon fibers effectively enhances the overall mechanical properties of the evaporator. This enhancement allows the material to maintain structural stability under substantial tensile and compressive deformations, thereby laying a solid foundation for the long‐term stable operation of the evaporator.

To investigate the evaporation performance of the evaporator prepared by the research institute, we constructed a system for validation (Figure S3). Experiments were conducted using standard solar cells to calibrate solar irradiance, as shown in Figure 4d, recording the changes in the water quality of CFCA, CFA, and CPA over time to explore the solar steam generation performance of different evaporators. In the absence of sunlight, pure water evaporated very slowly into the environment at a rate of 0.21 kg m^−2^ h^−1^, as illustrated in Figure 4f. Throughout the experiment, all calculated evaporation rates were adjusted by subtracting this value. Under simulated solar irradiance conditions of 1 kW m^−2^, the pure water evaporation rates of CFA and CPA were 3.15 and 3.03 kg m^−2^ h^−1^, respectively, while the evaporation rate of CFCA reached an astonishing 4.08 kg m^−2^ h^−1^(calculation methods in support information) [57]. This indicates that it possesses the highest evaporation rate. Figure 4e further confirms this finding, showing that CFCA achieves the highest temperature and the fastest temperature rise within the same time frame, indicating its superior photothermal conversion efficiency. Additionally, as illustrated in Figure 4f, under dark conditions, the evaporation rate of CFCA remains higher than that of CPA and CFA, suggesting that due to its unique internal simulated volcanic channels (Figure 3d,e), CFCA also exhibits commendable evaporation capability in the absence of light.

The evaporation performance of solar absorbers CPA, CFA, and CFCA under the same lighting conditions (Figure 4g) was evaluated. Under the irradiation of 1 kW m^−2^, the pure water evaporation rates of CPA, CFA, and CFCA were 3.03, 3.15, and 4.08 kg m^−2^ h^−1^, respectively, with their water evaporation efficiencies being 90.0%, 91.4%, and 95.8%. Under the same conditions, the evaporation rate and efficiency of CFCA were significantly higher than those of CPA and CFA, which can be attributed to its internally constructed simulated volcanic channel structure, effectively ensuring upward water transport and exhibiting a high water transport capability. In addition, the evaporation performance of CPA, CFA, and CFCA in simulated seawater solution (3.5 wt.% NaCl solution) was tested (Figure 4h). The evaporation rates of CPA, CFA, and CFCA calculated based on the formula were 2.75, 2.86, and 3.11 kg m^−2^ h^−1^, respectively, with evaporation efficiencies of 78.5%, 79.5%, and 80.1%, respectively. It is evident that in the simulated seawater solution, CFCA exhibits the highest evaporation rate and efficiency. This remarkable performance in saline water evaporation may stem from the unique structure of CFCA, where the Marangoni effect induced by the temperature gradient efficiently facilitates desalination to the periphery, thereby minimizing the blockage of water transport channels by salt crystals. CFCA demonstrates excellent performance in terms of light absorption, mechanical properties, evaporation rate, and efficiency (Figure 4i), showcasing the best overall performance.

### 3.4 Solar‐Driven Interfacial Water Evaporation of CFCA

**FIGURE 5:**
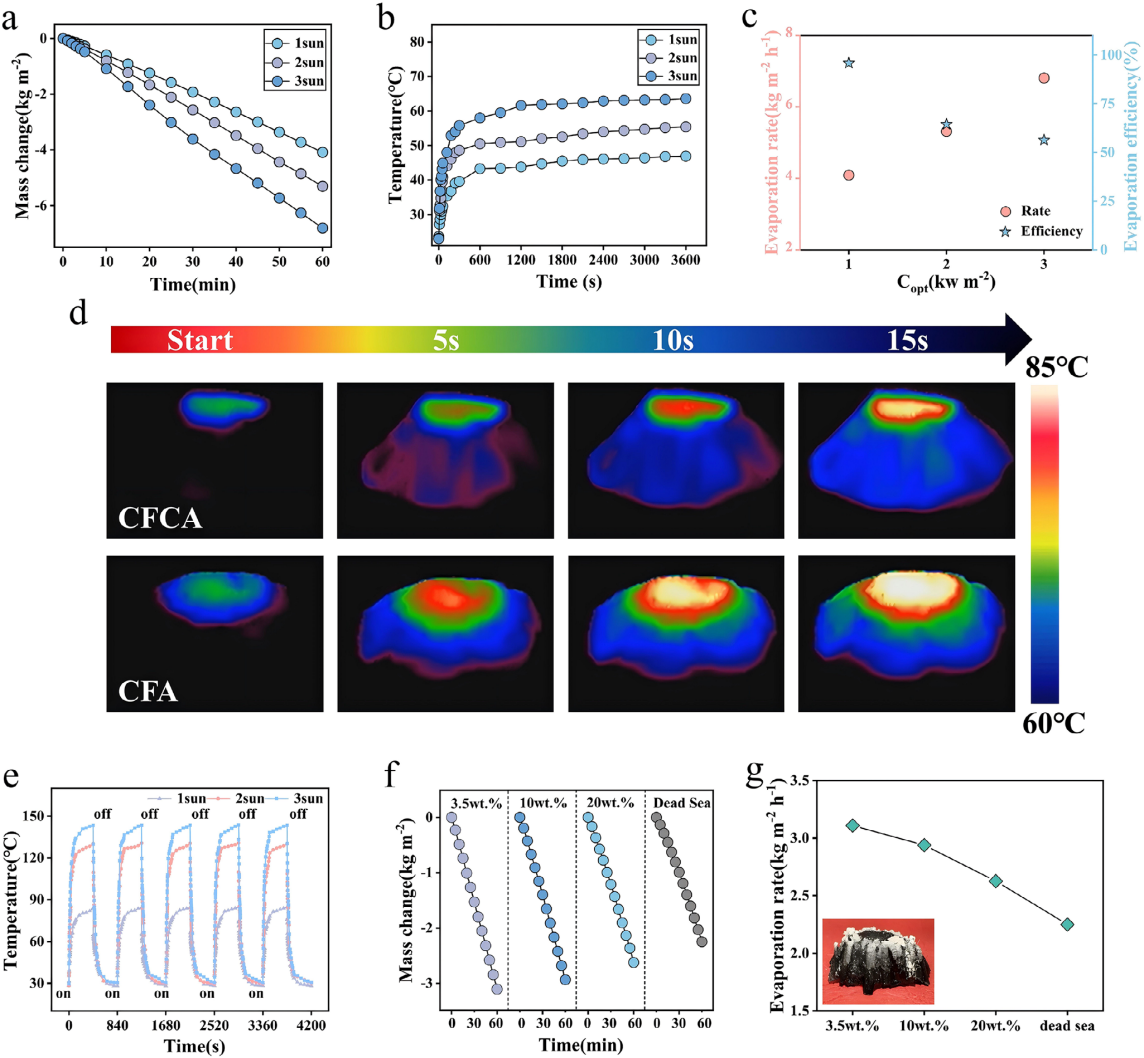
(a) Changes in water mass with solar exposure time at different optical concentrations for CFCA. (b) Surface temperature change under different optical concentrations for CFCA. (c) The evaporation performance of CFCA under different optical concentrations. (d) The infrared thermal images of different samples in the wet state. (e) Temperature cycling of CFCA in dry state at different optical concentrations. (f) The water mass variation over time for CFCA in varying concentrations of simulated seawater under 1 sun irradiation. (g) The evaporation rates of CFCA in varying concentrations of simulated seawater under 1 sun irradiation and the image of salt crystals precipitating in salt water by CFCA.

The evaporation performance of CFCA under different solar irradiation was studied. As shown in Figure 5a, the mass change of CFCA under simulated solar radiation conditions of 1, 2, and 3 kW m^−2^ significantly increases with the increase in light intensity, with mass changes of 4.08, 5.31, and 6.81 kg m^−2^ after 1 h, respectively. The mass of water evaporation rises with increasing light intensity, demonstrating that CFCA exhibits excellent photothermal sensitivity and light responsiveness. In Figure 5b, it can be observed that as the light intensity increases, the temperature of CFCA rises rapidly and remains constant after reaching a stable value, indicating that CFCA can efficiently convert light energy into thermal energy and exhibits good thermal stability under different light intensities. Figure 5c further indicates that with the increase in light intensity, the evaporation rate shows an upward trend, suggesting that CFCA has excellent photothermal conversion performance, with a maximum evaporation efficiency of approximately 95.8% under different light intensities, indicating its excellent evaporation stability and adaptability to varying light conditions in most regions.

To evaluate the photothermal performance of the evaporator, this study subjected CFCA and CFA to a simulated solar irradiation of 5 kW m^−2^ under wet conditions, and the surface temperature changes over time were recorded in real‐time using infrared thermography (Figure 5d). The experimental results indicate that the surface temperatures of both samples rapidly increased with the duration of illumination; however, their temperature distribution characteristics exhibited pronounced differences. The surface temperature of CFCA rose sharply from the initial temperature to approximately 85°C within 15 s, and the infrared thermography images clearly revealed a volcano‐like temperature distribution that closely followed the macroscopic geometry, with the highest temperature localized at the central region and a gradual decrease toward the periphery. This structured temperature profile originates from the synergistic heat‐storage and anisotropic heat‐redistribution effects enabled by the peripheral carbon‐fiber framework of CFCA. Owing to its high thermal conductivity and excellent thermal stability [63, 64], the carbon‐fiber network rapidly conducts and redistributes thermal energy along the periphery under illumination [65, 66]. As a result, heat is preferentially spread laterally and dissipated at the rim, which suppresses uncontrolled downward heat leakage into the bulk water and stabilizes a well‐defined center‐to‐edge temperature gradient across the evaporator. In contrast, although CFA also exhibits a rapid temperature rise under illumination, no distinct or geometry‐correlated temperature distribution is observed in the infrared images. Instead, CFA displays a diffuse thermal profile characterized by fluid‐like heat spreading, in which thermal energy continuously dissipates both laterally and downward into the surrounding environment. The absence of a continuous peripheral carbon‐fiber framework prevents effective lateral heat redistribution and stabilization of the thermal field, resulting in a relatively uniform temperature distribution without clear morphological features. Consequently, heat in CFA is more readily lost to the surroundings through convection and conduction, making it difficult to maintain a concentrated high‐temperature region at the evaporation interface. Further analysis indicates that the peripheral carbon‐fiber framework in CFCA plays a critical role in regulating heat transport pathways by combining efficient heat storage with directional lateral redistribution. This anisotropic heat management not only mitigates excessive thermal loss commonly observed in conventional photothermal materials, but also establishes a stable high‐temperature zone at the evaporation surface through controlled heat accumulation and spatial redistribution. Such a thermal environment accelerates interfacial heat transfer and phase change of water, thereby significantly enhancing photothermal conversion efficiency. In comparison, CFA lacks an effective structural component to regulate heat flow, leading to pronounced thermal dissipation and inferior photothermal performance. These results collectively demonstrate that the tailored heat‐management capability introduced by the peripheral carbon‐fiber framework is a key mechanism underlying the superior evaporation performance of CFCA and highlights the rationality of its structural design for efficient solar‐driven desalination.

Figure 5e shows the results of the cyclic light exposure tests of CFCA under different light intensities in a dry state. The material responds quickly to illumination at 1, 2, and 3 kW m^−2^, with a rapid increase in temperature, maintaining a stable heating and cooling cycle over five cycles. This demonstrates excellent photothermal responsiveness and cycling stability. This indicates that CFCA can maintain efficient photothermal conversion capabilities even after multiple light exposure cycles, providing assurance for its long‐term application in seawater desalination. Figure 5f illustrates the solar‐driven desalination performance of CFCA in simulated seawater at different concentrations (3.5%, 10%, 20%, and 27 wt.%). The mass change curves indicate that under sunlight, the evaporation rate of CFCA gradually decreases with increasing salt concentration. The evaporation rates of CFCA in 3.5%, 10%, 20%, and 27% NaCl solutions are 3.11, 2.94, 2.63, and 2.25 kg m^−2^ h^−1^, respectively (Figure 5g). This trend can be attributed to the precipitation and deposition of salt crystals at high salt concentrations, which may partially block the evaporation channels and affect the transport and evaporation of moisture. Figure 5g not only illustrates the evaporation rates at different salt concentrations but also presents the optical images of salt precipitation by CFCA in high‐concentration saline solutions. The results are consistent with those shown in Figure 5f, indicating that the higher the salt concentration, the lower the evaporation rate. The figure clearly shows the uniform precipitation of salt on the CFCA, suggesting that CFCA can effectively desalinate even in high‐salinity environments, with a relatively uniform distribution of salt crystals and no large‐scale aggregation of salt crystals. This further validates its stability and desalination capability in high‐salinity conditions.

### 3.5 The Desalination Mechanism and Salt Recovery of CFCA

**FIGURE 6:**
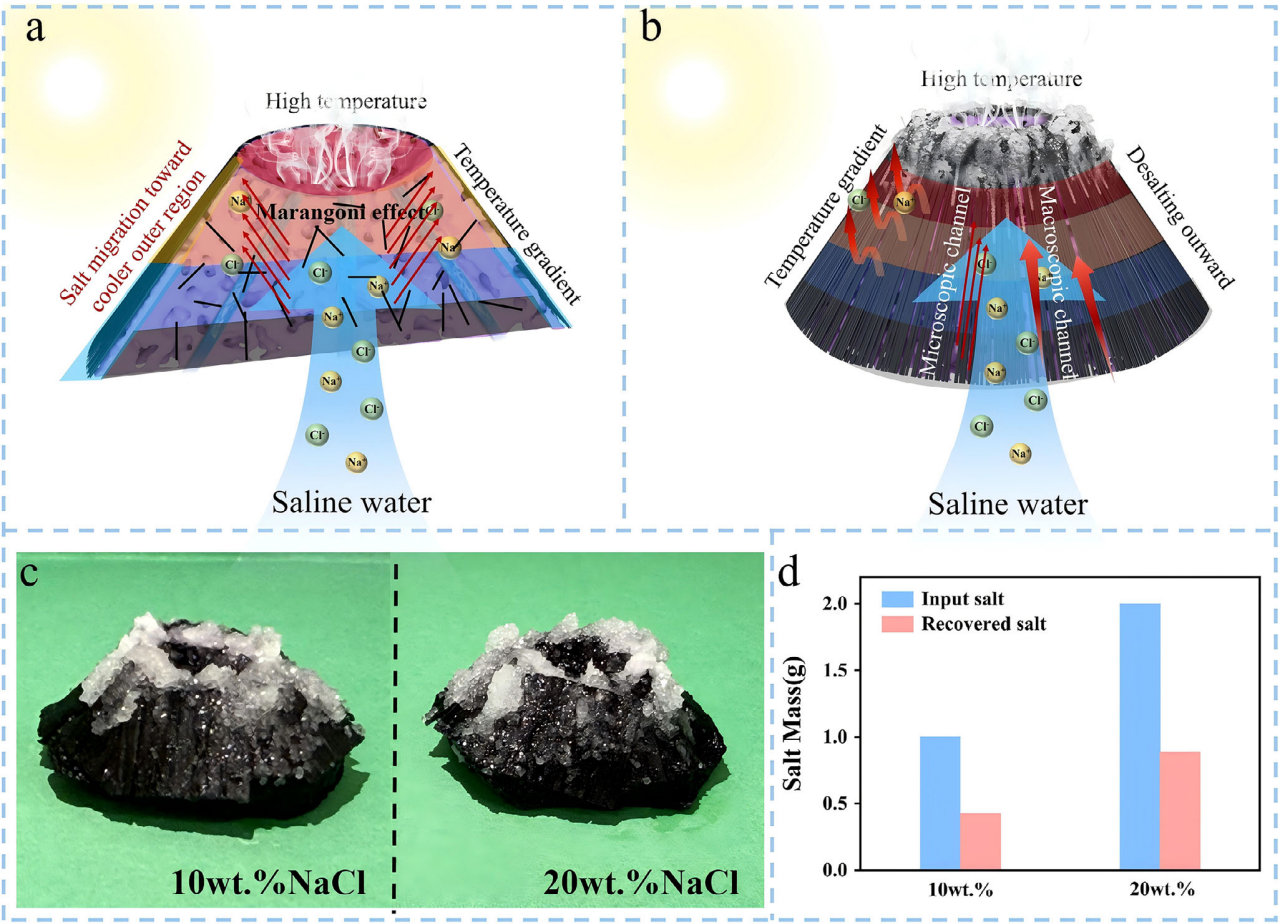
(a,b) The desalination mechanism of CFCA. (c) Real optical photos of CFCA after desalination in different concentrations of salt water. (d) The salt content of the desalinated water obtained by CFCA in different concentrations of salt solutions.

As shown in Figure 6a, the internal cross‐sectional structure of the CFCA and the overall structure of the CFCA depicted in Figure 6b are detailed as follows: The CFCA is designed with a core resembling a simulated volcanic configuration, featuring a unique bowl‐shaped crater structure at the top. Surrounding the top is a continuous ring of carbon fiber, while short‐cut carbon fibers are uniformly dispersed within, creating a dual carbon fiber network that is continuous on the outside and dispersed on the inside (Figure 6a). The porous and interconnected microscopic structure internally, along with the distribution characteristics of the carbon fibers, can be clearly observed in the cross‐sectional view (Figure 6b). During the seawater desalination process, when light interacts with the aerogel, the top bowl‐shaped volcanic structure rapidly heats up due to its excellent photothermal absorption performance. Simultaneously, the spatial confinement effect of the bowl‐shaped configuration concentrates heat in the volcanic crater region, leading to a stable temperature difference between the central evaporation zone and the peripheral regions of the aerogel, which is favorable for inducing Marangoni‐driven interfacial flow (Figures S4 and S5). Driven by the photothermal temperature gradient, the Marangoni effect continues to act on the seawater system within the aerogel, promoting the directional migration of salt ions (such as Na^+^, Cl^−^, etc.) toward the edge of the aerogel along the temperature gradient. Meanwhile, the surrounding continuous carbon fibers construct a permeable macroscopic transport channel, while the dispersed short‐cut carbon fibers within form interwoven microscopic pathways. The dual carbon fiber network works synergistically, not only reducing the interfacial resistance to salt ion migration but also establishing a multi‐level interconnected transport channel at both macroscopic and microscopic scales, significantly accelerating the accumulation process of salt ions toward the edge region (Figure 6a,b). As the illumination continues, the moisture in the top bowl‐shaped region of the aerogel rapidly evaporates under photothermal action, forming freshwater vapor and achieving the separation and collection of freshwater. Meanwhile, salt ions, which cannot volatilize with the vapor, are continuously concentrated and enriched in the multi‐stage migration channels, persistently migrating toward the edges of the aerogel. Ultimately, the concentrated salts are directed to precipitate from the edge of the bowl‐shaped volcanic crater at the top of the aerogel and the outer carbon fiber surrounding area (Figure 6b), without forming any coverage or deposition on the upper surface of the aerogel throughout the process. This effectively avoids the adverse effects of salt accumulation on the photothermal absorption efficiency of the aerogel and the seawater transport performance. Thus, this system constructs an efficient seawater desalination pathway through the photothermal response of the bowl‐shaped volcanic structure combined with the natural temperature gradient, the directional driving of salt ions by the Marangoni effect, and the synergistic multilevel transport of the dual carbon fiber network, achieving the simultaneous separation of freshwater and efficient discharge of salt. Cross‐sectional SEM–EDS mapping and line‐scan analyses further confirm the preferential enrichment of Na and Cl species in the peripheral region, providing direct spatial evidence for the edge‐directed salt crystallization behavior (Figure S6).

To verify the performance and recovery efficiency of CFCA in salt analysis, desalination and salt recovery experiments were conducted in 10 wt.% and 20 wt.% NaCl solutions. As shown in Figure 6c, salt crystals were observed to precipitate at the edges of the bowl‐shaped volcano at the top of the aerogel and in the outer region surrounded by carbon fibers, without forming a covering or deposition on the upper surface of the aerogel. This observation is consistent with the expected directional precipitation of salts discussed in the aforementioned mechanism analysis. The time‐resolved evolution of the salt crystallization process further supports this edge‐directed salt migration behavior, as shown in Figure S7. Further quantitative analysis of salt recovery efficiency was conducted (Figure 6d), and the results indicated that the salt recovery rate of CFCA was approximately 42.7% in a 10 wt.% NaCl solution and about 44.2% in a 20 wt.% NaCl solution. There is a high recovery efficiency that was still maintained at high salt concentrations. This demonstrates that CFCA possesses good salt crystallization and recovery capabilities across a wide concentration range. Therefore, in the process of seawater desalination, CFCA not only effectively enables the directed crystallization of salts, preventing their deposition on the surface of aerogels, but also maintains a high salt recovery efficiency under different salt concentrations, providing a promising technological solution for efficient seawater desalination and salt resource recovery. In addition, the CFCA evaporator can be readily cleaned after salt recovery and reused without causing a significant deterioration in evaporation performance (Figures S8 and S9).

### 3.6 Seawater Desalination Experiments and Outdoor Durability Testing

**FIGURE 7:**
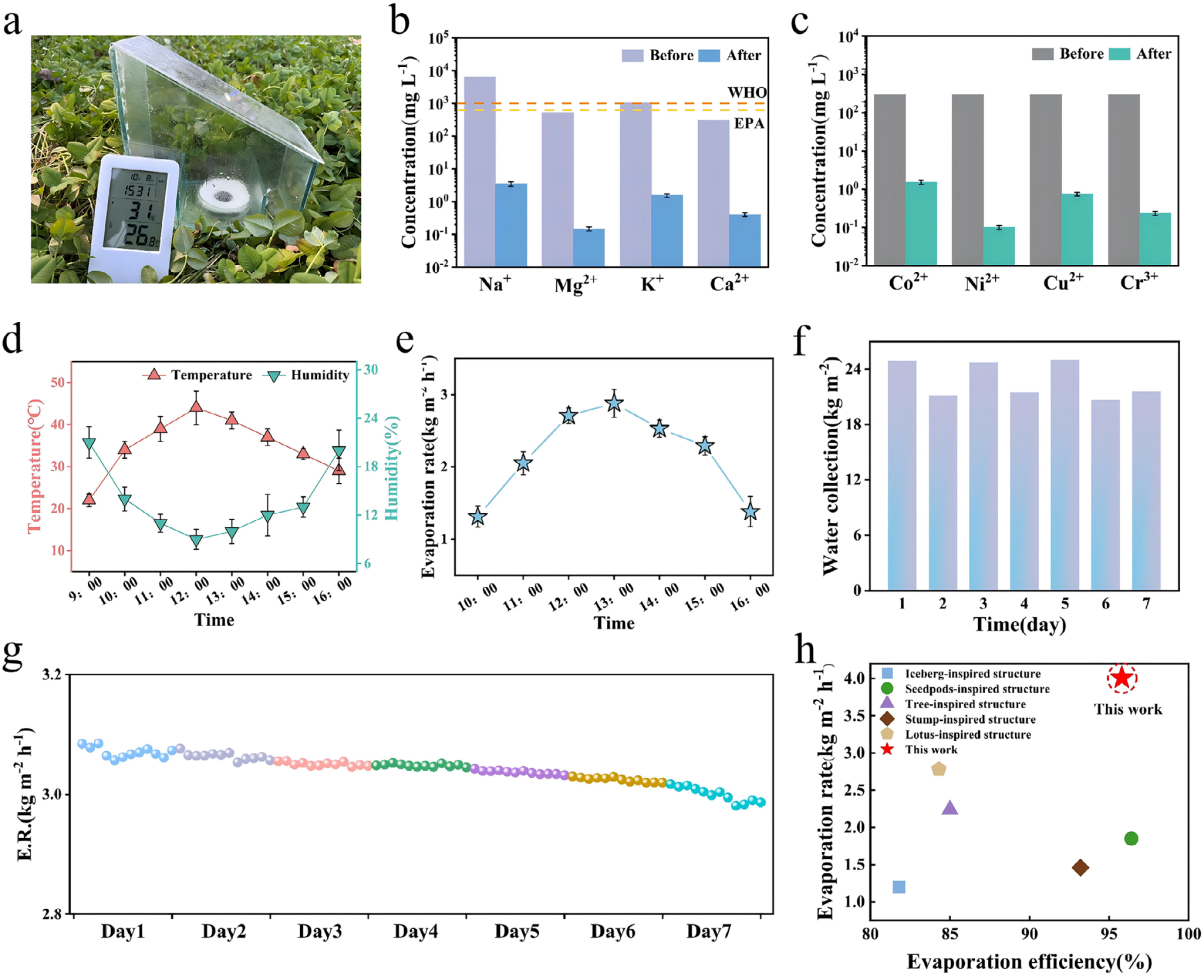
(a) Real optical image of the solar‐powered desalination unit. (b) The concentration of four major ions in the South Sea before and after the desalination process (the dashed lines represent the salinity standards for safety established by the WHO and the EPA, respectively). (c) The concentration of four heavy metal ions before and after seawater desalination. (d) Average ambient temperature and humidity during the 7‐day outdoor test. (e) Average evaporation rate during the 7‐day outdoor test. (f) Total water collection of CFCA under different weather conditions (7 days). (g) Long‐term evaporation rate of CFCA in 3.5 wt.% saltwater under simulated solar irradiation at 1 kW m^−2^. h Comparison of evaporation performance of CFCA with other structural evaporators.

Figure S10 shows the schematic diagram of the solar‐driven seawater desalination device, while Figure 7a presents an optical photograph of the device, which consists of an evaporation chamber and a condensation cover. Vapor is generated from the CFCA surface and then condenses into water on the condensation cover, subsequently flowing down along the walls of the evaporation chamber and collecting at the bottom partition. By analyzing the concentration changes of four major seawater ions in the South China Sea before and after desalination using CFCA, the actual desalination performance is assessed (Figure 7b). The concentrations of Na^+^, Mg^2+^, K^+^, and Ca^2+^ were significantly reduced from 6460, 532, 1044, and 312 mg L^−1^ to 3.52, 0.148, 1.532, and 0.4 mg L^−1^, respectively, far below the safety salinity limits established by the World Health Organization (WHO) and the United States Environmental Protection Agency (EPA). The purification effect of CFCA on real seawater has been validated. Further tests on the desalination of heavy metal solutions using CFCA showed a significant reduction in the total concentration of heavy metal ions after distillation, as illustrated in Figure 7c. The concentrations of Co^2+^, Ni^2+^, Cu^2+^, and Cr^3+^ were significantly reduced from 300 mg L^−1^ to 0.251, 0.261, 0.172, and 0.140 mg L^−1^, respectively. This reduction is attributed to the presence of numerous hydroxyl, carbonyl, and other oxygen‐containing functional groups on the surface of polyvinyl alcohol acetal and carbon fiber. The oxygen atoms in these functional groups contain lone pair electrons, which can interact weakly with the empty orbitals of metal ions, facilitating the adsorption of these ions from the solution onto the material surface and thereby lowering the ion concentration in the solution. Additionally, when these oxygen‐containing functional groups coordinate with heavy metal ions, they can form more stable chelate structures than ordinary coordination bonds. This strong binding force ensures that heavy metal ions are firmly fixed on the material surface, achieving efficient removal and effectively facilitating seawater desalination [38]. This indicates that CFCA not only exhibits excellent water purification performance for actual seawater but also further desalinate heavy metal solutions, providing technical support for more mature low‐energy seawater desalination methods.

Figure 7d,e show the average environmental temperature, humidity, and hourly evaporation rate from October 8 to October 14, 2024, between 9:00 and 16:00. The trend in evaporation rate aligns with the trend in environmental temperature. The results indicate that the CFCA can collect up to 25.02 kg m^−2^ of clean water (Figure 7f), with an average evaporation rate of 2.16 kg m^−2^ h^−1^. Although this evaporation rate is lower than the rate obtained under laboratory conditions (4.08 kg m^−2^ h^−1^), it still demonstrates an excellent evaporation rate. This difference is primarily influenced by a combination of factors such as temperature, humidity, solar radiation intensity, and light angle in the actual environment [67]. As shown in Figure 7g, the long‐term seawater desalination performance of CFCA was also demonstrated in a 3.5 wt.% NaCl solution, which has a salinity similar to seawater. A 12 h evaporation test was conducted under sunlight, followed by 12 h of exposure in darkness to simulate the alternation of day and night, lasting a total of 7 days. As illustrated in Figure 7g, during the 7‐day measurement, the evaporation rate slightly fluctuated from 3.08 to 2.98 kg m^−2^ h^−1^, while maintaining stability with an average of 3.00 kg m^−2^ h^−1^. This indicates the stable operation of CFCA in saline water over an extended period, confirming its efficient salt resistance and reliable long‐term performance. The solar evaporation performance of the CFCA inspired by volcanic structures was compared with previously reported evaporators under the same conditions inspired by different structures (Figure 7h) [50, 51, 52, 53, 68].

## 4 Conclusions

In this study, a volcano‐inspired dual‐carbon‐network aerogel evaporator was successfully fabricated via a facile sol–gel and freeze‐drying process. The unique architecture integrates hierarchical conduits composed of main, branch, and micropore channels. It also contains an internal network of dispersed short carbon fibers embedded in a carbon‐black aerogel matrix, together with a continuous outer carbon‐fiber shell. This integrated configuration not only provides efficient capillary water delivery and superior thermal confinement, but also endows the system with excellent mechanical stability and salt‐tolerant performance. Compared with conventional 2D planar or single‐network carbon aerogel evaporators, the volcano‐inspired 3D dual‐carbon architecture exhibits markedly enhanced interfacial heat localization and directional salt expulsion. The outer carbon‐fiber shell functions as a thermal barrier and conduction layer, while the inner dispersed short carbon fibers accelerate water replenishment and vapor escape through interconnected capillary pathways. As a result, the evaporator achieves an ultrahigh evaporation rate of 4.08 kg m^−2^ h^−1^ and a solar‐to‐vapor conversion efficiency of 95.8% under one‐sun illumination. Even under extreme salinity (20 wt.% NaCl), it sustains a high evaporation rate of 2.63 kg m^−2^ h^−1^ under one‐sun illumination with a 44.2% salt recovery efficiency, while maintaining long‐term stability in 3.5 wt.% brine for 7 days with an average rate of 3.00 kg m^−2^ h^−1^. The integrated design of water, heat, and salt transport, together with its facile fabrication strategy, offers a scalable route for sustainable solar desalination and recoverable salt crystallization.

## Conflicts of Interest

The authors declare no conflicts of interest.

## Supporting information




**Supporting File**: advs74823‐sup‐0001‐SuppMat.docx.

## Data Availability

The data that support the findings of this study are available from the corresponding author upon reasonable request.
