# Supplementary material for: Volcano‐Inspired Dual‐Carbon Network Aerogel for High‐Performance Solar Evaporation With Edge‐Directed Salt Crystallization and Recovery
Source: Adv Sci (Weinh). 2026 Mar 12;13(29):e74823. doi: 10.1002/advs.74823 (PMC13205595; doi:10.1002/advs.74823)
Supplement: Supplementary file 1 — Supporting File: advs74823‐sup‐0001‐SuppMat.docx. [file ADVS-13-e74823-s001.docx]

Supporting Information

Volcano-Inspired Dual-Carbon Network Aerogel for High-Performance Solar Evaporation with Edge-Directed Salt Crystallization and Recovery

Shuyue Feng, Yongpeng Wang^*^, Mengzhu Liu^*^, Xin Wang, Tao Jia, Haibo Zhang, Haoyue Wu, Yuan Xu, and Linghui Kong

**S. Feng, Y. Wang, M. Liu, X. Wang, H. Wu, Y. Xu, L Kong**
College of Materials Science and Engineering, Jilin University of Chemical Technology,
Jilin 132022, China
E-mail: wyp4889@163.com

**S. Feng**
College of Chemistry and Chemical Engineering, China University of Petroleum (East China),
Qingdao 266580, China

**T. Jia**
**Key Laboratory of Forest Plant Ecology, Ministry of Education, Engineering Research Center of Forest Bio-Preparation, College of Chemistry, Chemical Engineering and Resource Utilization, Northeast Forestry University**
Harbin 150040, China

**H. Zhang**
College of Chemistry, Jilin University,
Changchun 130012, China

1. Experimental Section

**1.1. Materials**

Conductive carbon black powder was supplied by the Jilin Renfei Technology Co., Ltd, China.Carbon fiber was supplied by the Jilin Guoxing Composite Material Co., Ltd,China. Poly(vinyl alcohol) (polymerization degree: 1700, alcoholysis degree: 88%) was purchased from Shanghai Aladdin Biochemical Technology Co.,Ltd, China. Glutaraldehyde (50% in H2O,analysis) was purchased from Shanghai Macklin Biochemical Co., Ltd, China. All of the above chemicals were of analytical grade and were directly used without any other treatment.

**1.****2. Characterization**

The morphologies of the samples were characterized using a field-emission scanning electron microscopy (FESEM) system (JEOL, JSM-7610F Plus, Japan) equipped with an energy-dispersive X-ray spectroscopy (EDS). Fourier transform infrared spectroscopy (FT-IR) (SHIMDZU, 1.50U1, Japan) was employed to identify the vibrations in the functional groups in the aerogels. The contact angle of our aerogels was tested using a contact angle meter ( POWEREACH, JC2000D2, China). The aerogels' light absorbance was measured from 300 to 1200 nm using a UV-vis Spectrometer (Tianjin Tuopu Instrument Co., Ltd., TP-760, China) with a slow reflection integrating sphere. The contents of metal ions in solution were measured using a handheld inductively coupled plasma emission spectrometer (PerkinElmer, Avio200, U.S.A.). The mechanical properties were tested and analyzed using a universal testing machine (Suzhou Kezhun Measurement and Control Co., Ltd, KZ-SSBC-500, China).

**1.3. Solar steam generation experiments**

Solar-driven water water evaporation experiments were carried out with a solar simulator (CEAULIGHT, CEL-PE300L-3A) to simulate solar flux ranging from 0 to 3.0 kW·m^-2^. The thermocouple power meter was utilized to monitor the optical power on the sample surface. Temperature fluctuations were observed on the upper surface of the CFCA using a thermal infrared camera (HIKMICRO, H16, China). The mass change due to water sorption was measured with a high-precision balance (0.1 mg accuracy). CFCAs were placed on polyethylene foam with a one-dimensional channel in order to evaluate their rate of evaporation.

**1.4.** **Optical measurements**

The absorption spectra of the devices were measured using the ultraviolet-visible spectroscopy. The absorption efficiency was calculated as, where A represents absorption efficiency, R represents reflection efficiency, and T represents transmission efficiency (). The solar absorption of CFCA was found to be approximately 98%.

**1.5. Calculation of energy efficiency**

The evaporation rate (v) and energy efficiency (η) were calculated via the following formula:^[1]^

$v=\frac{m}{st}$ (1)

where m is the mass loss of water during evaporation, s is the evaporation area of the samples, and t is the evaporation duration of the test.

$\eta=\frac{\mathop{V}\limits^{.} H_{LV}}{P_{0}C_{opt}}$ (2)

$\mathop{v}\limits^{.} =v_{light}-v_{dark}$ (3)

where v is the evaporation rate by subtracting the evaporation rate under dark conditions, P_0_ is the solar irradiation power of one sun (1 kW·m^-2^), and C_opt_ refers to the optical concentration on the absorber surface. H_LV_ is the equivalent evaporation enthalpy of the water in CFCA, which can be estimated by evaporating water under dark condition assuming identical energy input (U_in_).

$U_{in}=\Delta H_{vap}v_{0}=H_{LV}v_{g}$ (4)

where H_vap_ and v_0_ refers to evaporation enthalpy (2240J·g^-1^) and mass change of water (without hydrogel evaporator) (0.22 kg·m^-2^·h^-1^) under the dark condition, respectively. v_g_ is the mass change of CFCA within the same environmental condition.

1. **Supplementary Figures**


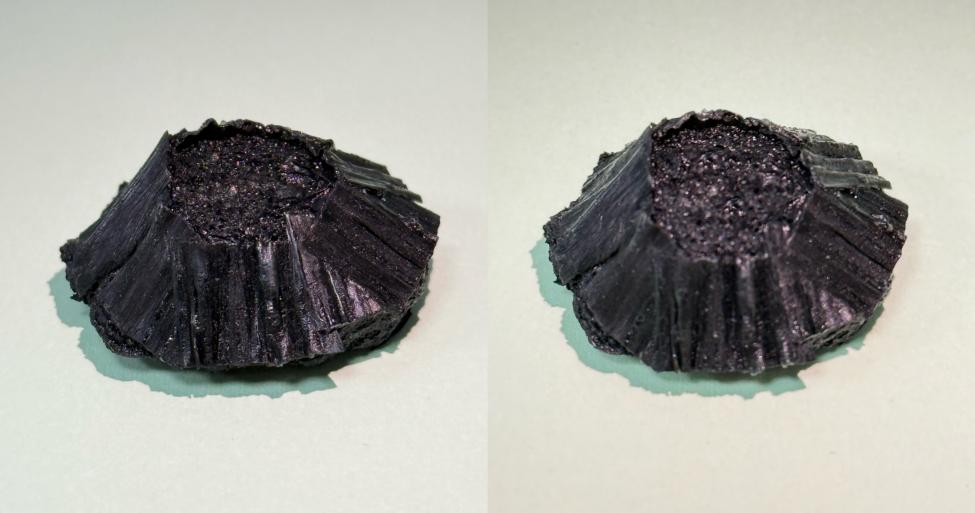


**Figure S1.** Real optical photographs of the as-fabricated CFCA evaporator.


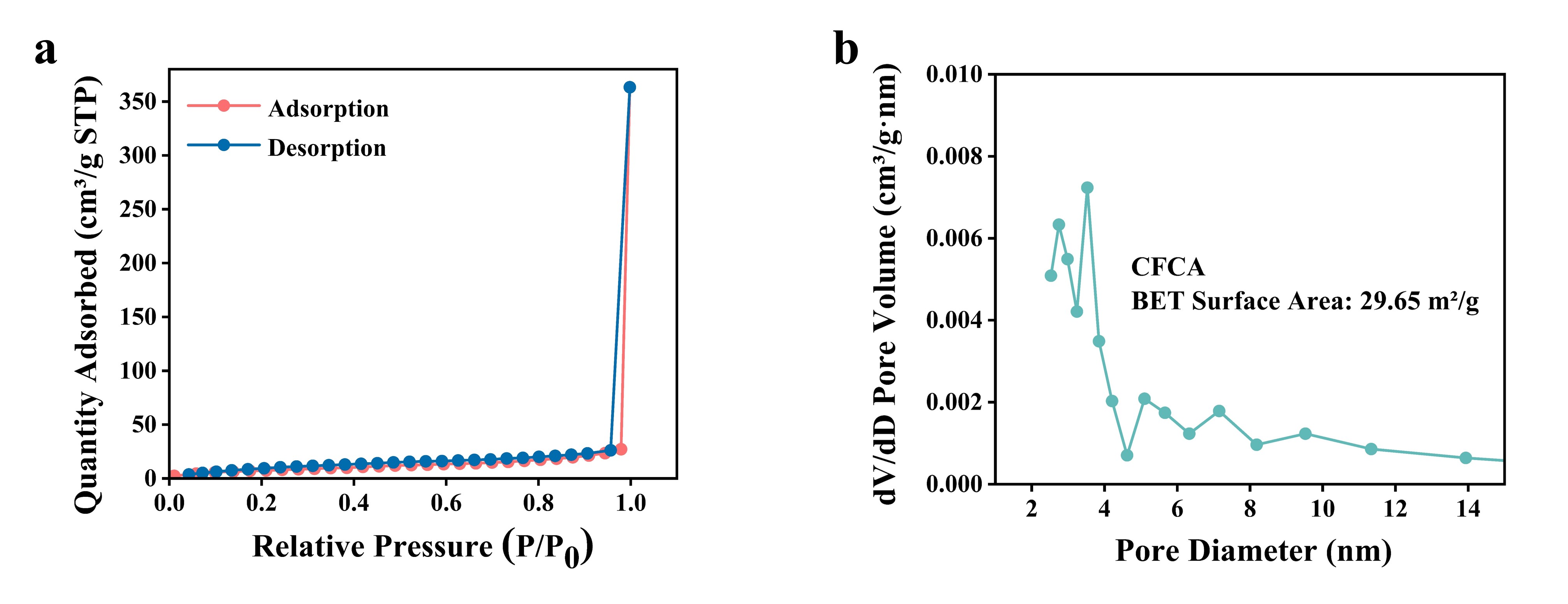


**Figure S2.** (a) Nitrogen adsorption–desorption isotherms of the CFCA sample at 77 K. (b) BJH pore size distribution of CFCA derived from the adsorption branch.


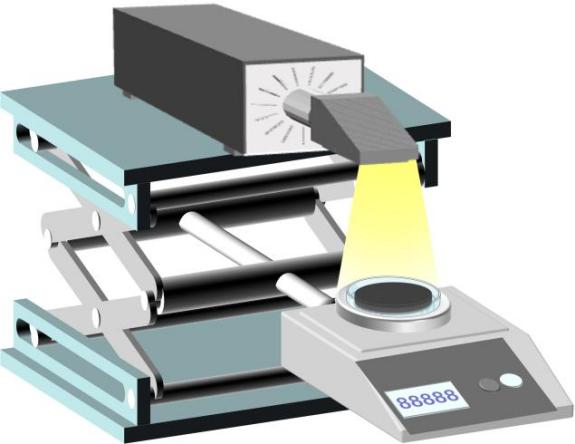


**Figure S3.** Schematic diagram of the evaporation system.


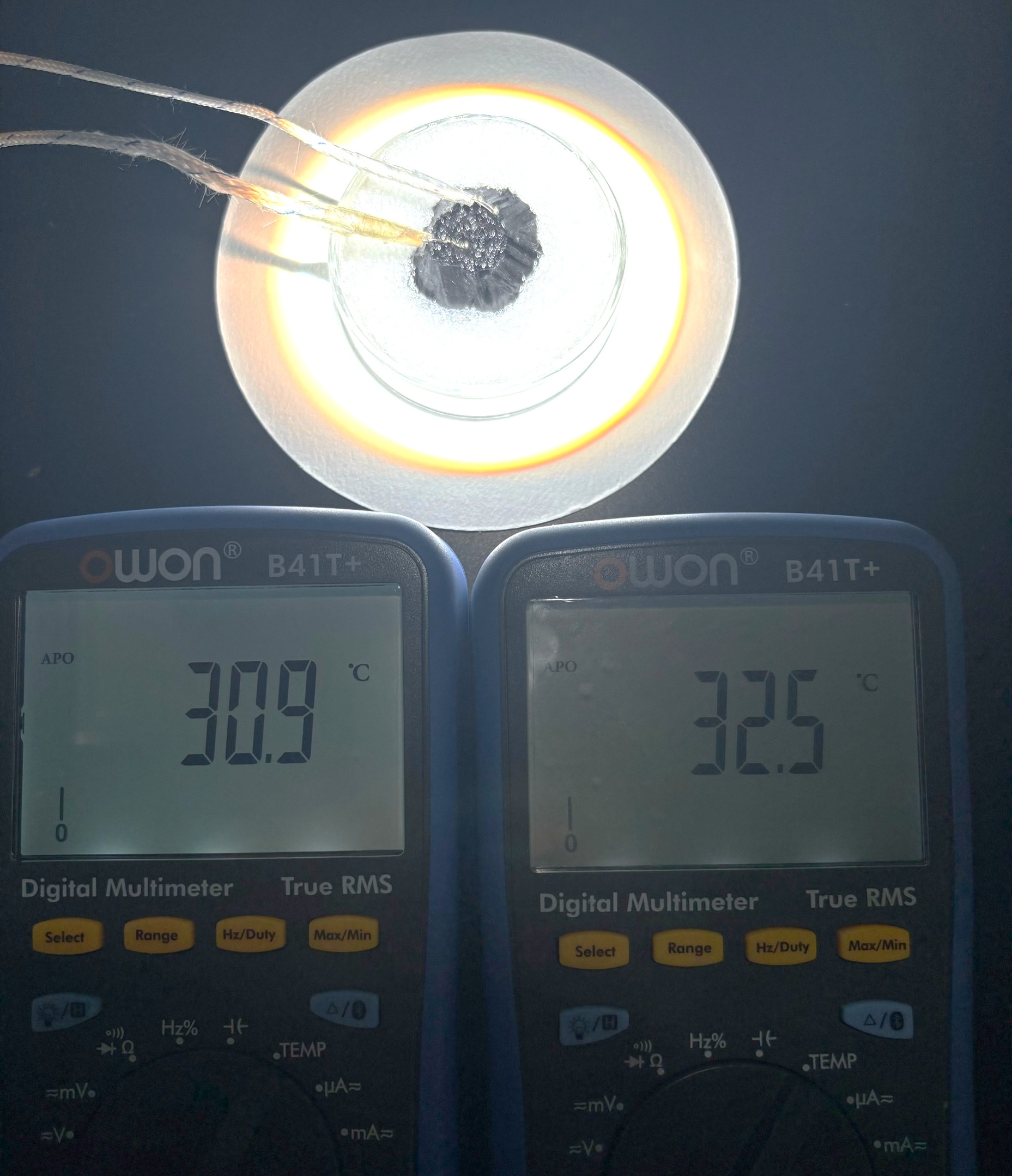


**Figure S4.** CFCA thermocouple test optical image.

**
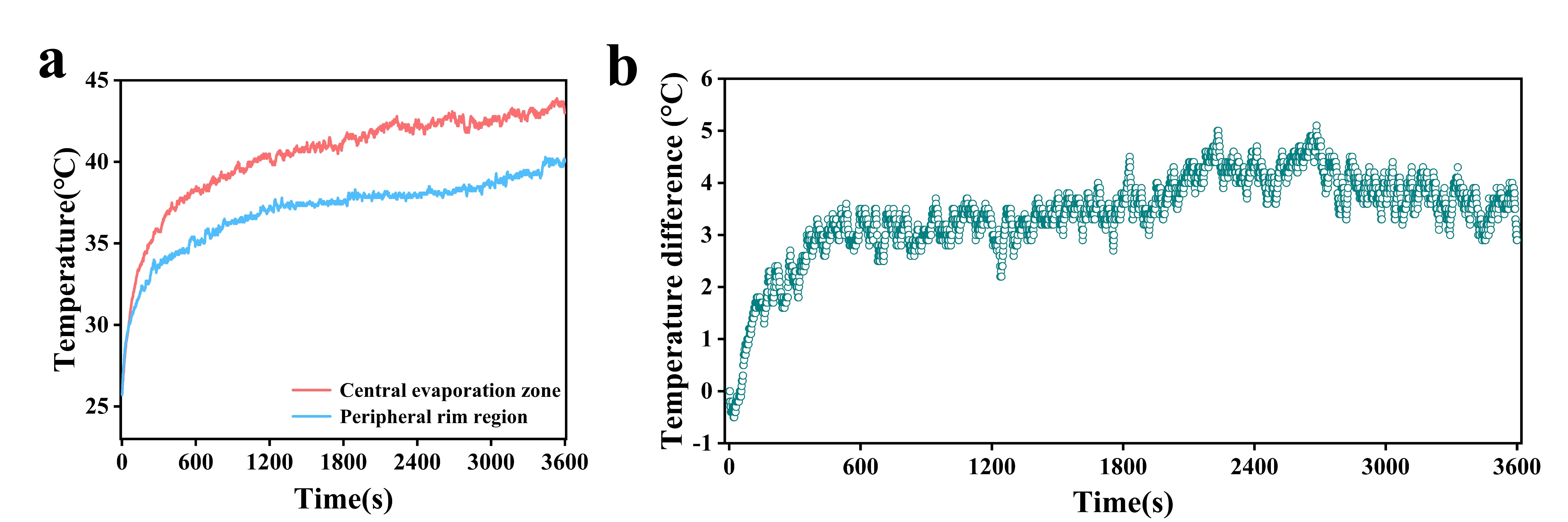
**

**Figure S5.** Time-dependent temperature profiles of the CFCA evaporator: (a) temperatures at the central evaporation zone and peripheral rim region; (b) corresponding temperature difference.


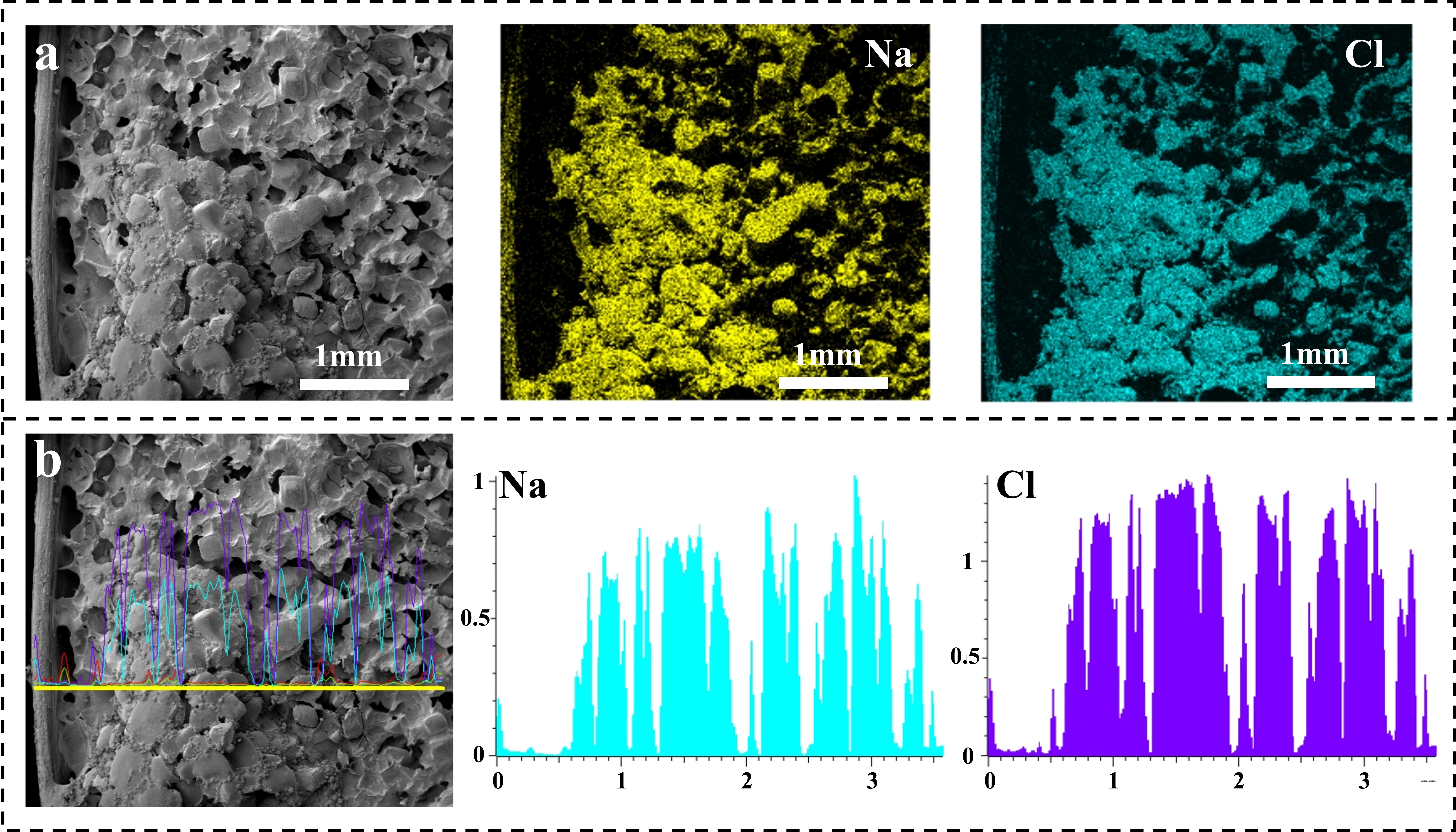


**Figure S6.** (a) Cross-sectional SEM image of the CFCA evaporator after saline evaporation and the corresponding EDS elemental mappings of Na and Cl in the peripheral region; (b) Cross-sectional EDS line-scan profiles of Na and Cl across the selected edge region of the CFCA evaporator.


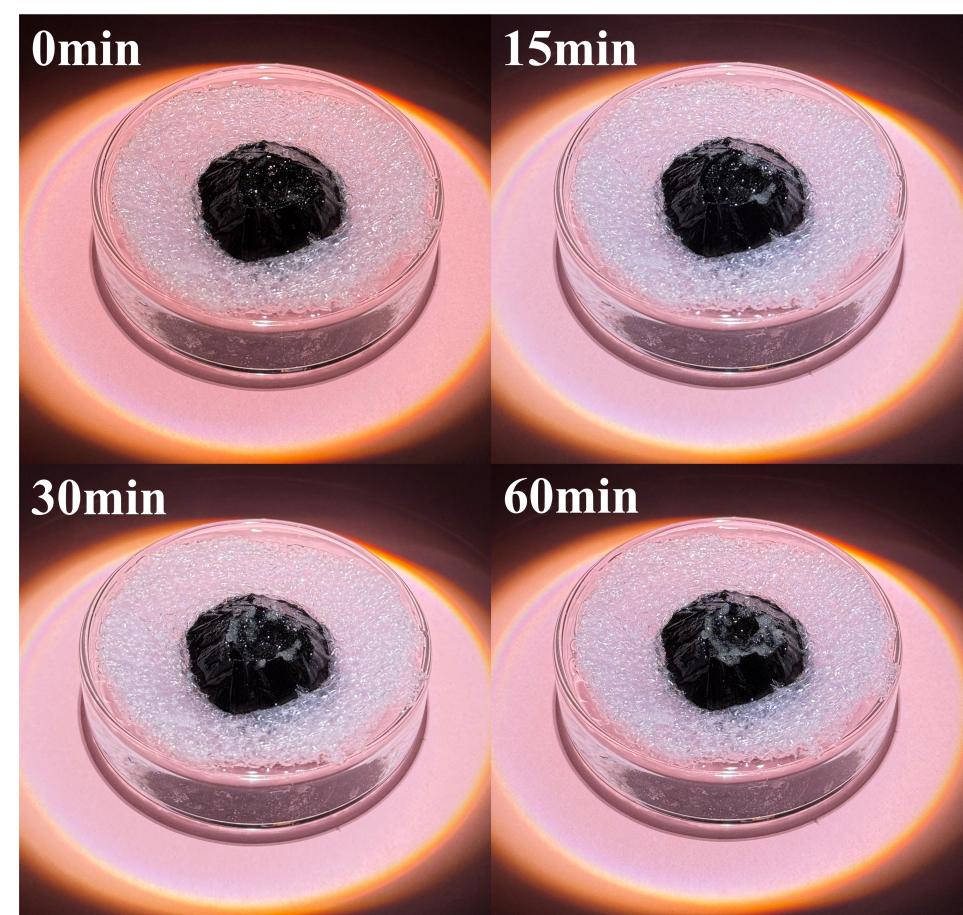


**Figure S7.** Optical images showing the evolution of salt crystallization on the CFCA evaporator during solar evaporation.


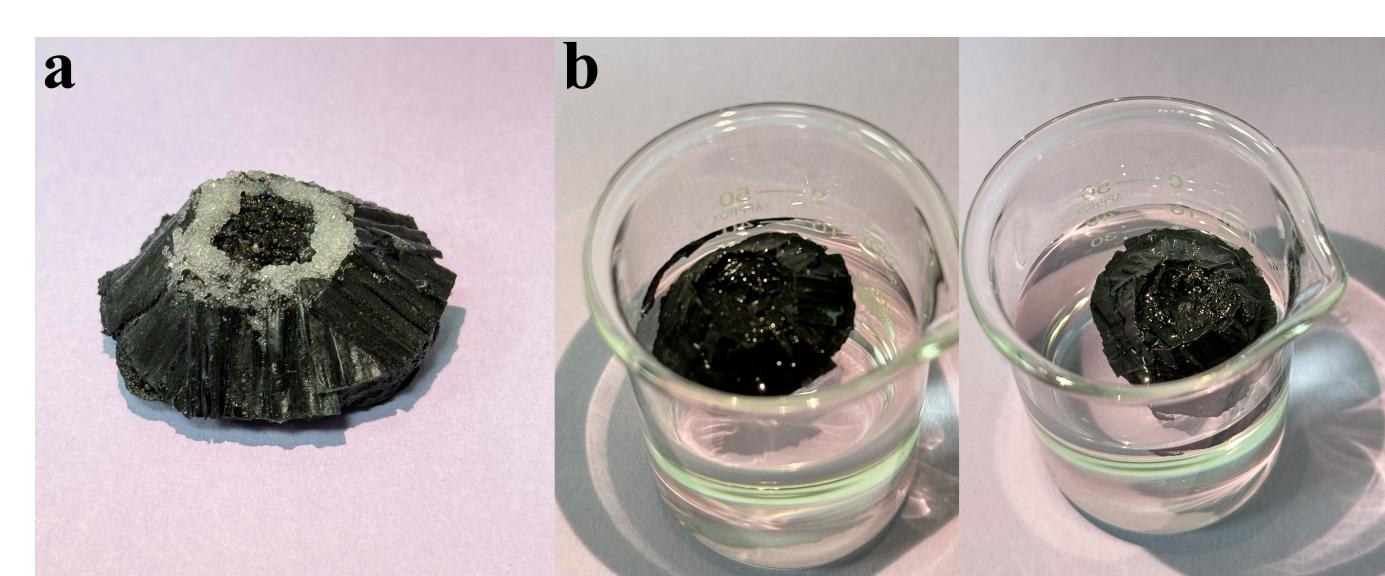


**Figure S8.** (a) Optical photograph of the CFCA evaporator after salt crystallization at the periphery following continuous evaporation in concentrated saline water. (b) Optical images of the CFCA evaporator immersed in deionized water during the cleaning process after salt recovery.


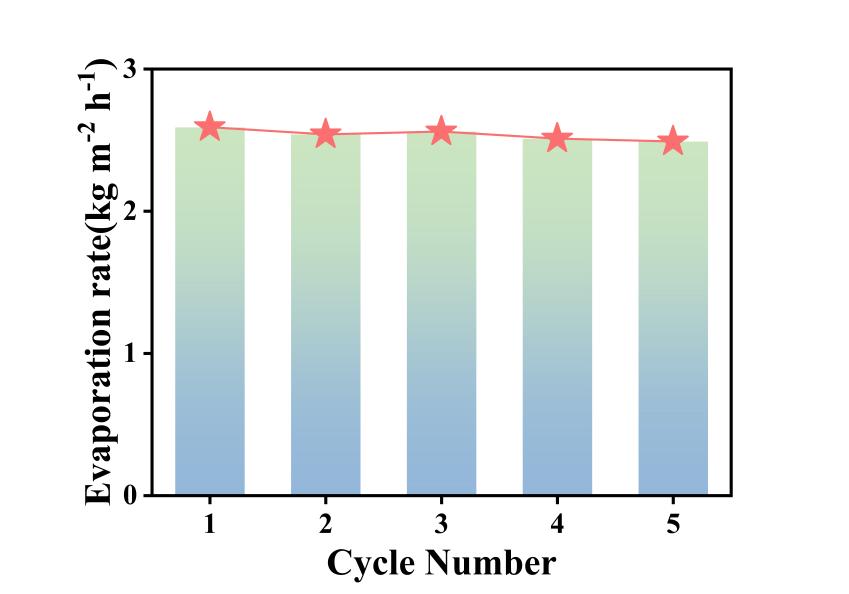


**Figure S9.** Evaporation rate of the CFCA evaporator over multiple reuse cycles.

**
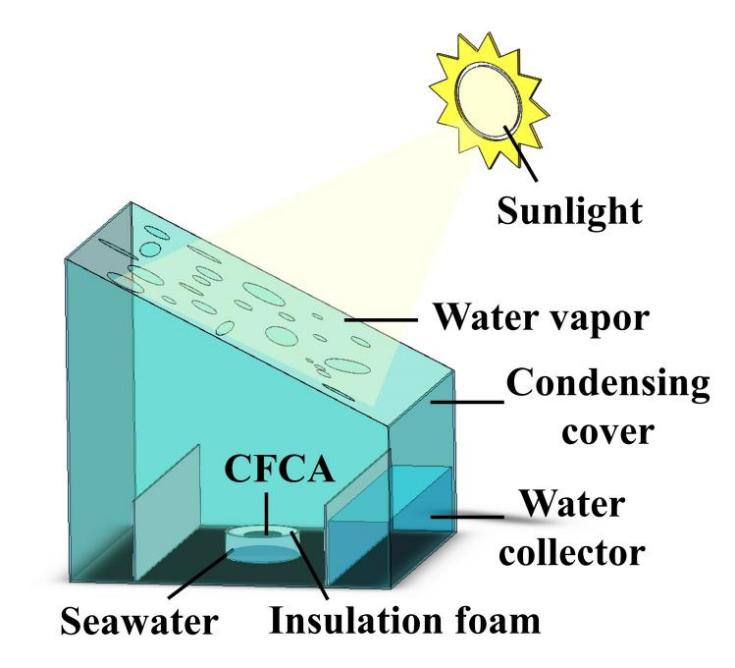
**

**Figure S10.**  Schematic illustration of the solar-powered desalination unit.

Supporting Information is available from the Wiley Online Library or from the author.

References

[1] H. Duan, M. Wang, Z. Zhang, J. Zhen, W. Lv, Journal of Environmental Chemical Engineering 2023, 11 (2), https://doi.org/10.1016/j.jece.2023.109295
